# Cancer in Women over 50 Years of Age: A Focus on Smoking

**DOI:** 10.3390/cancers7010450

**Published:** 2015-03-17

**Authors:** Luiz Francisco Baccaro, Délio Marques Conde, Lúcia Costa-Paiva, Vanessa de Souza Santos Machado, Aarão Mendes Pinto-Neto

**Affiliations:** 1 Department of Gynecology, State University of Campinas, Rua Alexander Fleming, 101, Cidade Universitária Zeferino Vaz, Campinas, São Paulo 13.083-881, Brazil; 2 Breast Clinic, Hospital for Maternal and Child Healthcare, Goiânia, Goiás 74.125-120, Brazil; E-Mail: delioconde@gmail.com; 3 Department of Gynecology, State University of Campinas, Campinas, São Paulo 13.083-881, Brazil; E-Mails: paivaepaiva@uol.com.br (L.C.-P.); vssm80@gmail.com (V.S.S.M.); aarao@unicamp.br (A.M.P.-N.)

**Keywords:** neoplasms, menopause, risk factors, life style, smoking

## Abstract

The increase in life expectancy worldwide has resulted in a greater prevalence of chronic non-communicable diseases. This study aims to evaluate the prevalence and factors associated with the occurrence of cancer among Brazilian women over the age of 50. A cross-sectional study with 622 women over the age of 50 was performed using a population survey. The outcome variable was the occurrence of a malignant tumor in any location. The independent variables were sociodemographic characteristics, self-perception of health, health-related habits and morbidities. Statistical analysis was carried out using the chi-square test and Poisson regression. The mean age of the women was 64.1 years. The prevalence of cancer was 6.8%. The main sites of occurrence of malignant tumors were the breast (31.9%), colorectal (12.7%) and skin (12.7%). In the final statistical model, the only factor associated with cancer was smoking > 15 cigarettes/day either currently or in the past: PR 2.03 (95% CI 1.06–3.89). The results have improved understanding of the prevalence and factors associated with cancer in Brazilian women aged 50 years or more. They should be encouraged to maintain a healthy lifestyle and pay particular attention to modifiable risk factors such as smoking.

## 1. Introduction

While population aging is a global phenomenon, its effects are more pronounced in developed countries than in low-income countries. In Brazil, average female life expectancy at birth is 77.3 years; which compares with figures of 57.8 and 82.4 years for low- and high-income countries, respectively [1]. Furthermore, previous work has shown that, on average, at age 50 years a Brazilian woman will live an additional 31 years [2]. This increase in life expectancy, both in Brazil and other countries, has resulted in an increase in the prevalence of chronic non-communicable diseases (NCDs), including malignant neoplasms. However, longer life expectancy is only one explanation for the increase in the incidence of cancer. In high-income countries, reported incidence is higher than in low-income countries partly as a result of more widespread access to screening and diagnostic procedures for cancer [3].

The climacteric, or menopause, is a period during which women go through several hormonal and psychological changes [4,5]. During menopause, women often have greater concern for their health and perceive they are more likely to develop chronic diseases. This is therefore an opportune period during which to target interventions to promote positive lifestyle changes, in addition to screening programs for NCDs such as cancer [3].

Expanding the evidence base regarding the epidemiology and risk factors associated with malignancies affecting postmenopausal women is instrumental for the development of public health policies and interventions; both for planning screening programs and for designing and implementing campaigns to promote the adoption of healthier lifestyles [6]. We therefore conducted a study to determine the prevalence of malignancies, and their associated risk factors, among women over 50 years of age living in the city of Campinas (SP, Brazil).

## 2. Methods

### 2.1. Sampling Methods

Our study used population-level cross-sectional data collected as part of “Health conditions in women over 50 years of age”. This was a population based study in Campinas-SP, Brazil using a survey that was conducted from May 10 to October 21, 2011. According to the Brazilian Institute of Geography and Statistics (IBGE), the population of Campinas in 2007 was 1,039,000, of whom approximately 545,000 were women. Of these women around 131,800 were over 50 years of age. Given that this was a survey on multimorbidity, and that hypertension is reported to be the most prevalent disease in women in Brazil and in developed countries, we used the prevalence of hypertension, estimated at 56.3%, to calculate an appropriate sample size. We assumed a type 1 error rate (alpha) of 5% and a margin of error of 5%. The resulting sample size estimate was then increased by a further 10% to offset against non-response. This calculation resulted in a target sample size of 657 women to be invited to participate in the study.

Participants were recruited from 66 randomly selected, official census sectors in the city of Campinas from a numbered list provided by IBGE, using simple random sampling. Our classification of these census sectors, each of which had clearly defined geographic boundaries, was based on an official census of the city of Campinas conducted in 2000. All census sectors containing at least 10 women aged 50 years or over were included in the random selection process. Those sectors in which there were fewer than 10 women in this age group were grouped with the consecutively numbered neighboring sector. Research assistants were provided with maps of each census area and visited odd-numbered street addresses in a systematic fashion. The researchers then verified whether there were any woman aged 50 years or over at each address, and invited all those who met the eligibility criteria to participate in the study. If two or more women who met the inclusion criteria lived at the same address, only one was selected to participate based on alphabetical order of names. The women who agreed to participate were interviewed, either in-person or by telephone, by trained research staff from CEMICAMP (Center for Research and Control of Maternal & Child Diseases of Campinas, Campinas, Brazil). This process was repeated until 10 eligible women were recruited from each census sector. If the target number of respondents was not achieved, then researchers visited those addresses that were not initially considered for inclusion. Of a total of 721 women who were invited to participate in the study, 99 (13.7%) refused to participate, with the most common stated reason for non-response being a lack of time to complete the questionnaire. This resulted in a final sample of 622 women. Participation in the study was voluntary and all subjects gave written informed consent before being interviewed. Ethical approval for this study was granted by the Research Ethics Committee of UNICAMP under the protocol number 1012/2010.

### 2.2. Inclusion Criteria

Women over 50 years of age residing in the city of Campinas were eligible for inclusion in the study. Potential participants were excluded if they were considered unable to respond to the questionnaire for any reason, including illness, personal commitments, or unavailability at the time of survey. Additionally, we also excluded those diagnosed with cognitive disabilities or dementia.

### 2.3. Outcome Variable

The outcome variable for the present study was the presence or absence of a malignant tumor in any part of the body. This classification was made using the following question: “Has a doctor ever said that you have cancer or a malignant tumor?” Participants were able to answer “yes”, “no”, or “do not know”. Those participants who responded “yes” to this question were then asked to identify the location of the cancer or malignant tumor.

### 2.4. Questionnaire

The questionnaire used as part of the present study was based on three other pre-existing questionnaires; two developed in Brazil [7,8] and another developed in the United States [9]. It was divided into five sections covering sociodemographic variables, health behaviors, self-reported perception of health, functional capacity, and existing health problems. All data collected by the survey were based on self-reporting.

## 3. Statistical Analysis

We initially made a simple descriptive analysis to characterize the frequency distributions of the occurrence of different types of cancer in our study sample. A bivariate analysis of the outcome variable against each independent variable was then performed using a chi-square test [10]. Finally, we analyzed the survey data using a multiple Poisson regression model with covariates selected using a forward procedure [11]. In the latter analysis, we calculated the prevalence ratios (PR) for each covariate in addition to their associated 95% confidence intervals (95% CI). Statistical significance was set at the 5% level, and clustering (by census sector) in our sampling design was accounted for in both the bivariate and multivariate analyses. All analyses were carried out using SPSS version 20 (IBM Corp, Armonk, NY, USA) and Stata version 7 (StataCorp, College Station, TX, USA).

## 4. Results

A total of 622 women responded to our questionnaire. While the mean age of our sample was 64.1 years, 70.4% had eight or fewer years of education, 52.2% were not living with a partner, 70.4% were white, 53.6% reported a family income of R$1500 or less, 37.9% had a BMI between 25 and 29.99, 36.3% were currently or previous smokers, 15% reported regular alcohol consumption, and 36.2% exercised weekly. Some relevant clinical and sociodemographic characteristics are shown in Table 1.

**Table 1:** Clinical and sociodemographic characteristics (n = 622).

Characteristic	%
**Schooling**	
≤8 years	70.4
>8 years	29.6
**Skin color**	
White	70.4
Non-white	29.6
**Marital Status**	
With partner	47.8
Without partner	52.2
**Family income**	
≤R$ 1500	53.6
>R$ 1500	46.4
**BMI**	
<25	37.2
25–29.9	37.9
≥30	24.9
**Smoking**	
Yes (currently or in the past)	36.3
No	63.7
**Alcohol consumption**	
Yes	15.0
No	85.0
**Weekly physical exercise**	
Yes	36.2
No	63.8
**Hypertension**	
Yes	55.9
No	44.1
**Diabetes**	
Yes	22.7
No	77.3
**Multimorbidity**	
Yes	58.2
No	41.8

The prevalence of cancer in this population sample was 6.8%. The most commonly reported sites of malignant tumors were the breast (31.9%), the colo-rectum (12.7%), and the skin (12.7%) (Table 2).

**Table 2:** Prevalence and location of cancer in women aged ≥50 years (n = 622).

	n	%
Cancer		
Yes	42	6.8
No	580	93.2
Total	622	100.0
Location *		
Breast	15	31.9
Colorectal	6	12.7
Skin	6	12.7
Head and Neck	4	8.5
Lung	3	6.3
Uterus	3	6.3
Thyroid	3	6.3
Ovary	2	4.2
Stomach	2	4.2
Other	3	6.3

* Includes women with cancer in more than one location.

Our bivariate analysis identified associations between the likelihood of reporting any type of cancer and smoking more than 15 cigarettes per day either currently or in the past (*p* = 0.03), hospitalization in the past year (*p* < 0.01), use of antiulcer drugs (*p* = 0.04), and having private medical insurance (*p* = 0.04). Our results also showed a negative association between the outcome and time since participants’ most recent medical consultation (*p* < 0.01). Statistically significant associations are shown in Table 3.

Finally, the results of our Poisson regression analysis indicated a positive association between an increased likelihood of reporting any type of cancer and smoking more than 15 cigarettes per day either currently or in the past (RP: 2.03, 95% CI: 1.06–3.89, *p* = 0.03).

**Table 3:** Distribution of the women in accordance with the occurrence of cancer and variables of health care (n = 622).

Variable	Cancer			
	Yes	No	(n)	*p* *
Number of cigarettes smoked per day				0.03
0–15	6.0	94.0	(516)	
>15	12.2	87.8	(90)	
Hospitalization in the past year				<0.01
Yes	16.0	84.0	(81)	
No	5.4	94.6	(540)	
Time since last medical consultation (months)				<0.01
≤5	8.4	91.6	(474)	
>5	1.6	98.4	(127)	
Use of antiulcer drugs				0.04
Yes	13.5	86.5	(52)	
No	6.2	93.8	(568)	
Private medical insurance				0.04
Yes	9.0	91.0	(300)	
No	4.7	95.3	(322)	

* Chi-square test considering the cluster sampling plan: census sector (primary sampling unit).

## 5. Discussion

In 2008, 63% of all deaths worldwide were attributable to NCDs, with the majority of these as a result of four groups of diseases, namely diabetes, cardiovascular diseases, chronic respiratory disease, and cancer [12]. In that same year, the World Health Organization (WHO) published an action plan to promote measures to reduce the impact of NCDs on the global population [6]. Since then, greater emphasis has been placed on the prevention and control of these diseases. Among the objectives of the WHO action plan are the promotion of research into NCDs and the improvement of epidemiologic monitoring [6].

The prevalence of cancer in our sample population of women over 50 years of age (6.8%) was relatively high. This figure contrasts with the results of the “National Research by Household Sample” (PNAD) carried out in 2008, which found a prevalence of cancer of 0.61% among Brazilian women of all ages. The prevalence of malignancies among both men and women aged 50–59 years was 1.22%, rising to 3.57% among those aged 80 years or over. While this same study found that the prevalence of cancer in women was 20% higher than in men, there was a 33% increase in the prevalence of malignancies in PNAD 2008 when compared with PNAD 2003 [13]. Another comparable cohort study that aims to investigate the burden of NCDs in the Brazilian population, the Longitudinal Study of Adult Health (ELSA-Brazil), was initiated in 2008 and surveys adults aged 35–74 years. This study found a 5% baseline prevalence of cancer among women, similar to that found in the present study [14]. In our study, the most common sites of occurrence of cancer were the breast (31.9%), the colo-rectum (12.7%), and the skin (12.7%). This pattern of distribution is in accordance with the most recent estimates of the incidence of different types of malignant neoplasms among Brazilian women, which also show that non-melanoma skin cancer, breast cancer, and colorectal cancer are most frequently reported [15]. With the development of more effective therapeutic methods and an increase in survival rates, it is expected that the prevalence of each type of cancer will more closely resemble the pattern of its incidence over time.

In the present study, smoking more than 15 cigarettes (or around one pack) daily, either at the time of the survey or in the past, was found to be the only risk factor independently associated with a diagnosis of any type of cancer. Women who reported smoking 16 or more cigarettes per day were twice as likely to report a diagnosis of cancer. In our population sample, 36.3% of respondents were current smokers or had been smokers in the past. Among those who smoked, approximately 40% smoked at least one pack of cigarettes a day.

Smoking is the single most common cause of cancer worldwide [16]. Among those types of cancer reported by the women in our study, there are proven associations between smoking and colorectal, head and neck, lung, cervical, ovarian, and stomach cancer [16]. However, the association between smoking and breast cancer is yet to be fully understood. Although some large cohort studies shown a weak association [17,18], a recent study among Canadian women found no association between smoking and the risk of breast cancer [19].

One of the goals of the WHO action plan is to promote actions to reduce exposure to modifiable risk factors associated with the development of NCDs, including tobacco use [6]. The Ministry of Health of Brazil, through its “Surveillance of Risk and Protective Factors for Chronic Diseases through Telephone Survey (VIGITEL)” program, has tracked the prevalence of smoking among the Brazilian population [20]. In 2013, the estimated smoking prevalence among women aged over 18 years was 8.6%, while 18.9% of women were ex-smokers. The same study found a significant reduction in the prevalence of smoking since 2006 when this figure was 12.4%. There was also a reduction in the proportion of women smoking more than 20 cigarettes a day from 3.2% in 2006 to 2.4% in 2013. The number of women that reported passive exposure to tobacco smoke in the homes also decreased significantly between 2006 and 2013, from 13.4% to 10.7% [20]. This reduction in smoking may be because of the impact of public education campaigns aimed at increasing awareness of the health harms of smoking, and government interventions such as banning indoor smoking and increasing the sale price of tobacco though taxation [21]. In the present study, however, both the smoking prevalence and the proportion of ex-smokers were relatively high. This was likely owing to the fact that our survey only included women over 50 years of age. Nonetheless, it is expected that, over time, the prevalence of smoking among women would also decrease among this age group.

Our study had some limitations, however. First, the cross-sectional nature of our study prevented us from establishing cause-and-effect relationships between the exposure and outcome variables. Second, all variables were based on self-reporting by participants. However, given that this was a population-based study, any errors would have resulted equally in either an increase or a decrease in the diagnosis of the conditions investigated. Finally, the present study did not aim to identify the specific characteristics of each risk factor, but rather to identify the prevalence and factors associated with the occurrence of cancer in climacteric and postmenopausal women in general.

The climacteric and post-menopausal periods provide a window of opportunity for clinicians to conduct interventions for the screening and prevention of NCDs. Although the majority of patients may not experience disease symptoms, many women, including smokers, will consult a gynecologist during this period. Gynecologists can therefore play a major role, not only in tracking the development of neoplasms associated with smoking such as cancers of the lung, head and neck, and gastrointestinal tract (which are often not taken into account during a typical gynecological visit), but also in health promotion and in providing targeted advice for smoking cessation. They can therefore intervene in a meaningful way to prevent the development of malignancies and other NCDs, which represent the leading causes of mortality and decreased quality of life after the menopause.

## 6. Conclusions

The results of the present study show that the prevalence of any type of cancer in this population sample was 6.8%. The only factor associated with an increased likelihood of reporting any type of cancer was smoking more than 15 cigarettes per day either currently or in the past.

## Appendix: Independent Variables

Variables covered by the survey included age in years, years of education (up to eight years, more than eight years), marital status (with a partner, without a partner), race (white, non-white), monthly income (up to R$ 1,500.00, more than R$ 1,500.00), current body mass index (BMI) (less than 25 or greater than or equal to 25), smoking status (never smoker, past smoker or current smoker), number of cigarettes smoked per day (up to 15, 16, or more), current alcohol consumption (yes, no), frequency of alcohol consumption (never or less than once a week, once a week or more), weekly engagement in physical exercise (yes, no), weekly frequency of physical exercise (never, once or twice weekly, three times per week or more), hospitalization in the past year (yes, no), time since last medical consultation (up to 5 months, 6 months or more), use of any medication prescribed by a physician (yes, no), use of any medication acting on the central nervous system (yes, no), use of any medication for treatment of menopausal symptoms (yes, no), use of any anti-rheumatologic drug (yes, no), use of any antihypertensive medication (yes, no), use of any antilipidemic medication (yes, no), use of antidiabetic medications (yes, no), use of cardiorespiratory medications (yes, no), use of thyroid hormones (yes, no), use of antiulcer drugs (yes, no), analgesic use (yes or no), use of alternative therapies (yes, no), private medical insurance coverage (yes, no), cessation of menstruation (more than a year ago, no), problems maintaining balance while walking (yes, occasionally, or never), problems maintaining balance while taking a bath, dressing, or going down stairs (occasionally or never), self-rated perception of health (good or very good, fair or poor, or very poor), difficulties in feeding, taking a bath, or going to the toilet (unable or with significant difficulty, with little or no difficulty), ability to run, lift heavy objects or engage in sports or physically demanding work (unable or with significant difficulty, with little or no difficulty), ability to push a table or perform household chores (unable or with significant difficulty, with little or no difficulty), ability to climb stairs (unable or with significant difficulty, with little or no difficulty), ability to crouch or kneel down (unable or with significant difficulty, with little or no difficulty), ability to walk 100 m (unable or with significant difficulty, with little or no difficulty), ability to walk more than one km (unable or with significant difficulty, with little or no difficulty), diabetes (yes, no), osteoarthritis (yes, no), hypertension (yes, no); history of heart attacks (yes, no), history of stroke (yes, no), bronchitis or asthma (yes, no), emphysema (yes, no), osteoporosis (yes, no), bone fracture after 50 years of age (yes, no), glaucoma (yes, no), cataracts (yes, no), use of glasses or contact lenses (yes, no), ability to see well (yes, no or ”more or less”), use of hearing aids (yes, no), ability to hear well (yes, no or “more or less”), urinary incontinence (yes, no), engagement in regular sexual activity (yes, no), and number of morbidities (up to one, two, or more).
